# One Guest or Two? A Crystallographic and Solution Study of Guest Binding in a Cubic Coordination Cage

**DOI:** 10.1002/chem.201905499

**Published:** 2020-02-06

**Authors:** Christopher G. P. Taylor, Stephen P. Argent, Michael D. Ludden, Jerico R. Piper, Cristina Mozaceanu, Sarah A. Barnett, Michael D. Ward

**Affiliations:** ^1^ Department of Chemistry University of Warwick Coventry CV4 7AL UK; ^2^ Diamond Light Source Ltd., Diamond House Harwell Science and Innovation Campus Didcot, Oxfordshire OX11 0DE UK

**Keywords:** binding constants, coordination cage, host–guest chemistry, supramolecular chemistry, X-ray crystallography

## Abstract

A crystallographic investigation of a series of host–guest complexes in which small‐molecule organic guests occupy the central cavity of an approximately cubic M_8_L_12_ coordination cage has revealed some unexpected behaviour. Whilst some guests form 1:1 **H⋅G** complexes as we have seen before, an extensive family of bicyclic guests—including some substituted coumarins and various saturated analogues—form 1:2 **H⋅G_2_** complexes in the solid state, despite the fact that solution titrations are consistent with 1:1 complex formation, and the combined volume of the pair of guests significantly exceeds the Rebek 55±9 % packing for optimal guest binding, with packing coefficients of up to 87 %. Re‐examination of solution titration data for guest binding in two cases showed that, although conventional fluorescence titrations are consistent with 1:1 binding model, alternative forms of analysis—Job plot and an NMR titration—at higher concentrations do provide evidence for 1:2 **H⋅G_2_** complex formation. The observation of guests binding in pairs in some cases opens new possibilities for altered reactivity of bound guests, and also highlights the recently articulated difficulties associated with determining stoichiometry of supramolecular complexes in solution.

## Graphical abstract

**What a bind**: Despite solution titrations showing binding of one guest inside the cavity of an octanuclear cubic coordination cage, in the solid‐state many guests (such as coumarin, illustrated) bind as pairs with up to 87 % of cavity volume being occupied—amongst the highest packing coefficients known. With this knowledge from crystallography, evidence for formation of **H⋅G_2_** species can be found in solution under forcing conditions.

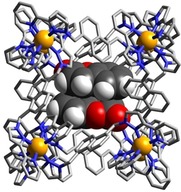

## Introduction

The ability of self‐assembled coordination cages to bind small‐molecule guests in their central cavities remains a highly popular area of investigation in the general field of supramolecular chemistry^1^, ^2^, ^3^ because of its relevance to a wide range of potential functions.^4^ As part of our ongoing investigations into the self‐assembly and host–guest chemistry of a family of such cages we have performed many quantitative studies on the binding of small‐molecule guests in the central cavities of our family of cages in solution.^5^, ^6^, ^7^, ^8^, ^9^, ^10^

**Scheme 1:**
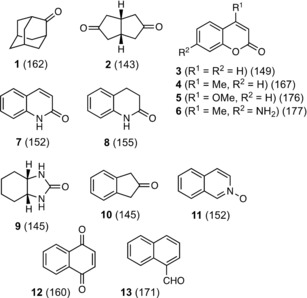
The guest molecules studied in this work. Numbers in parentheses are molecular volumes in Å^3^, calculated using Spartan.

**Figure 1:**
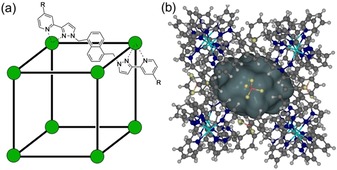
The host cages [Co_8_L_12_]^16+^, abbreviated as **H** (R=H; soluble in MeCN) and **H^w^** (R=CH_2_OH; soluble in water). (a) A sketch emphasising the cubic array of Co^II^ ions and the disposition of a bridging ligand; (b) a view showing the cavity space (volume 409 Å^3^).

Most of our work in this area has focussed on the octanuclear, approximately cubic, coordination cages [M_8_L_12_]X_16_ (Scheme 1) where M=Co^II^ or Cd^II^, and L is a bridging ligand containing two chelating pyrazolyl‐pyridine units at either end of a spacer that contains a 1,5‐naphthalene‐diyl group.^5^ The substituents on the ligand L help to control solubility: the unsubstituted ligand L (giving host cage **H**, Figure 1) affords cage assemblies that are soluble in polar organic solvents,^6^ whereas the ligand L^w^ produces isostructural cages (denoted **H^w^**, Figure 1) whose exterior coating of hydroxyl groups helps to provide solubility in water.^6^ Whilst we have found evidence for guest binding in the cage cavity of **H** in MeCN, driven by formation of weak hydrogen‐bonds between the guest and the cage interior surface,^7^ far stronger and more widespread binding of guests occurs in water due to the magnitude of the hydrophobic effect which ensures that hydrophobic guests of appropriate shape and size can bind with association constants of up to 10^8^ m ^−1^.^6^, ^8^, ^9^ In some cases this binding has resulted in efficient catalysis of reactions on the bound guest.^9^

Clearly, a detailed understanding of guest binding and the factors underpinning it are essential to underpin work on the properties of such host–guest systems. Guest binding is normally quantified by solution titration methods, often by ^1^H NMR spectroscopy, or by fluorescence spectroscopy if either cage or guest is fluorescent and this fluorescence changes on guest binding. In our recent work with **H** and **H^w^**,^3^, ^5^, ^6^, ^7^, ^8^, ^9^ in essentially all cases where a standard binding curve is obtained from a spectroscopic titration the data can be fit to a 1:1 binding isotherm to extract a single association constant for guest binding. On the basis of a large number of known binding constants from guests of various shapes and sizes we were able to develop a predictive algorithm, using molecular docking software, which could predict binding constants of guests in water with enough accuracy to be genuinely useful in identifying new guest targets.8c The only exception to 1:1 binding behaviour in solution that we have observed to this general pattern was with the relatively small guest dimethyl methylphosphonate (DMMP), whose binding curve, obtained from fluorescence quenching and NMR titration experiments, fitted better to a 1:2 host:guest (**H⋅G_2_**) complex.^10^ In this case the crystal structure of the cage/guest complex did show the presence of two DMMP guests in the cavity involved with weak hydrogen‐bonding interactions with two binding pockets on the interior surface. Apart from these cases however our measurements of guest binding constants by titrations have been consistent with formation of 1:1 **H⋅G** complexes and we have generally assumed this to be the case.

We report in this paper the results of a detailed structural investigation of complexes based on our cubic host **H**, with a wide range of guests, which substantially extends our understanding of the possibilities available for complex formation. In particular, the results of the combined crystallographic and solution binding study highlight important differences between the stoichiometry of a series of cage/guest complexes in solution and in the solid state. Of course there is nothing new in the idea that what is observed in a crystal structure is not necessarily the same as what happens in dilute solution, but the crystallographic studies reported here provide some quite unexpected results about possibilities for guest binding that nicely complement our observations from solution studies. The recent critical focus on methods used to determine the stoichiometry of supramolecular complexes (e.g. Job plots) and the erroneous conclusions that can be easily reached from careless application of unjustified assumptions makes this study particularly timely.^11^, ^12^

## Results and Discussion

### Background and previous work: Choice of guests

The series of guests that we investigated for binding in the cavity of cages **H/H^w^** is shown in Scheme 1. We investigated many of these a while ago during our early quantitative studies on cage/guest binding in different solvents. Two important results from these binding studies were that (i) these guests illustrated 1:1 binding behaviour in MeCN (**H**) or aqueous (**H^w^**) solution as shown by NMR or fluorescence titrations;^5^, ^6^, ^7^, ^8^ and (ii) in MeCN guest binding is in part driven by a hydrogen‐bonding interaction between an electron rich part of the guest (e.g. a carbonyl or pyridine‐*N*‐oxide O atom) and a collection of convergent CH bonds (arising from methylene CH_2_ and naphthyl CH units) on the cage interior surface, which lie close to a metal ion and are therefore in a region of positive electrostatic potential.^7^, ^13^ We estimated that the collection of CH bonds in these H‐bond donor pockets—there are two such pockets at opposite ends of the long diagonal of the cage superstructure—was comparable to a phenol group in terms of its overall hydrogen‐bond donor strength.^7^, ^13^ In the solid state, small guests such as DMMP or solvent molecules clearly show this hydrogen‐bonding interaction between the two guests and the H‐bond donor sites on the host interior surface.^5^, ^10^ Significantly, although this H‐bonding interaction between a bound guest and the cage interior surface is not a driving force for guest binding in aqueous solution, as it is weaker than the effects of solvation when host and guest are separated, it still serves to orient the guest once it is bound in the cavity.8a

Several examples of cage/guest complexes with larger guests that we have crystallographically characterised all demonstrate the same general behaviour, with the two H‐bond donor sites on the cage interior surface providing an anchoring point for H‐bond acceptor sites of the guest which help to position and orient the guest in the cavity. Thus the carbonyl group of a range of bulky aliphatic ketone guests is always anchored in this way,8a, 8d as is the carboxylate terminus of adamantane‐1‐carboxylate.8b When a single guest contains two H‐bond accepting functional groups with the correct separation it can span the cavity diagonally and interact with both H‐bond donor sites simultaneously, as we saw recently using 1,2,4,5‐tetracyanobenzene as a guest.8e

### Methodology for preparation of cage/guest crystals

We prepared the cage/guest complexes for crystallographic studies in this paper using the “crystalline sponge“ method,^14^, ^15^ famously recently popularised in supramolecular chemistry by the work of Fujita's group.^14^ This involved treating pre‐formed crystals of host **H** with guests which could be taken up into the crystals without loss of crystallinity. This has proven to be a far more reliable method than growing crystals of the cage from solution in the presence of guest which usually afforded crystals of the guest‐free cage containing only solvent molecules. Good quality crystals of [Co_8_L_12_](BF_4_)_16_ (**H**) can be prepared from a solvothermal reaction of Co(BF_4_)_2_ with the edge‐bridging ligand L (2:3 ratio) in MeOH followed by slow cooling,^5^ and these are robust enough to survive removal from the mother liquor and treatment with possible guests for hours or days, either as neat oils or concentrated solutions in various solvents. We have found that this method provides around a 1 in 3 chance of successfully obtaining a structure of a cage/guest complex, with the remaining data collections showing that guest was not taken up, resulting just in a structure of the cage with (usually disordered) solvent molecules in the cavity; or, in some cases, disorder of the guest that was too severe to model. When coupled to the high‐throughput capacity of synchrotron facilities with automated sample changing and 20‐minute data collections this allowed a large number of successful cage/guest structures to be determined quickly.

### 1:1 Cage/guest complexes with mono‐functional or bifunctional guests

In this section we describe two new examples of 1:1 **H⋅G** complexes, both of which are aliphatic ketones but with either one (guest **1**) or two (guest **2**) ketone units for anchoring to the H‐bonding sites on the cage interior surface.

**Figure 2:**
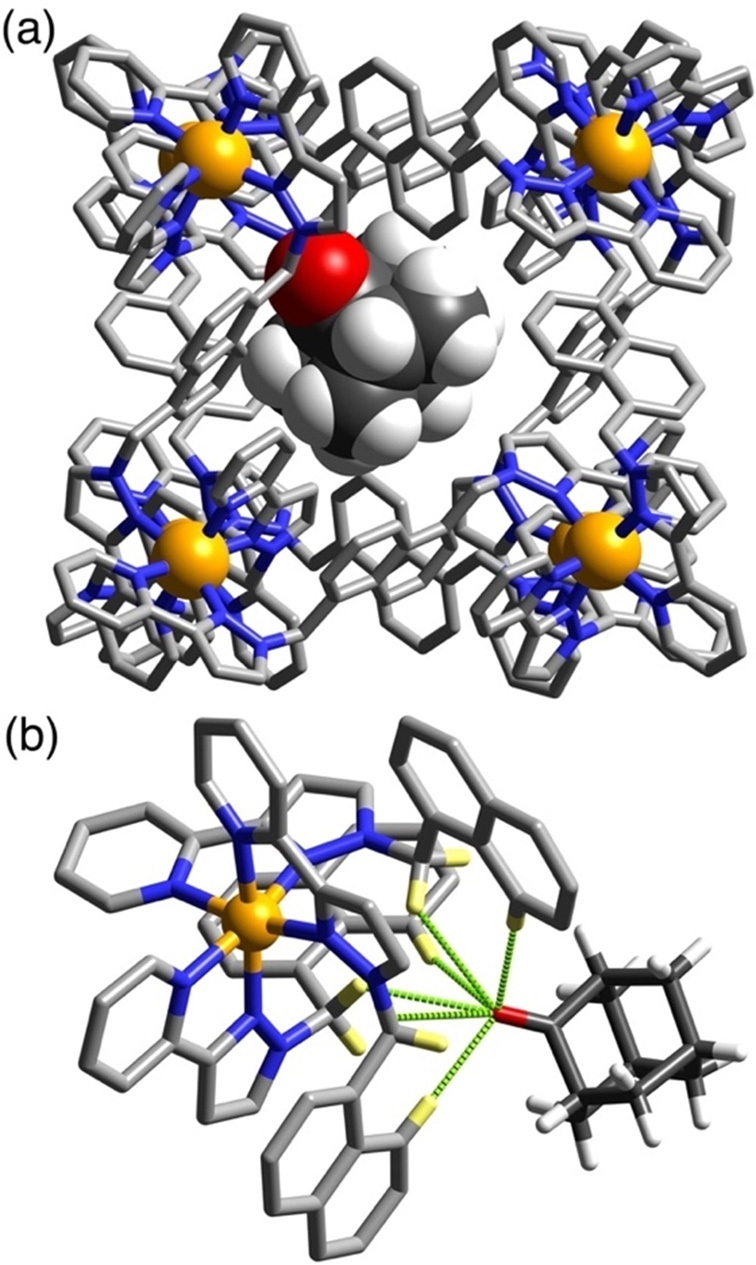
(a) Structure of the complex **H⋅1** with the host cage **H** in wireframe but the guest **1** shown space‐filling. A fractional‐occupancy water molecule also present in the cavity is not shown. (b) H‐bonding interactions in the structure of the **H⋅1** complex between the carbonyl unit of the guest and the convergent set of CH protons on the host (CH⋅⋅⋅O distances, 2.46–2.98 Å).

In Figure 2 is the structure of the complex **H⋅1**. The pseudo‐spherical skeleton of this guest is a good shape match for the host cavity and its molecular volume (162 Å^3^) is about 40 % of the volume of the host cavity (409 Å^3^, using a 1.2 Å sphere as the probe; see Figure 1 b) so there should be no steric issues: Rebek showed that a guest volume of around 55 % of the host cavity volume tends to give optimal binding in solution,^16^ and our own work has supported that.8a The carbonyl group, on the basis of previous experience, is expected to provide an anchoring point for the guest to one of the two H‐bond donor sites in the cavity,8a, 8d and so it proved. We can see that the C=O group is oriented such that the oxygen atom interacts with the collection of convergent CH protons in the cage binding pocket, with several CH⋅⋅⋅O contacts from naphthyl CH and methylene CH_2_ protons having H⋅⋅⋅O separations in the range 2.46–2.98 Å; individually weak but cumulatively clearly significant (Figure 2 b). There is only room for one 2‐adamantanone guest in the cavity of **H**; leaving the second binding pocket, at the opposite end of the cavity diagonal, with room to accommodate a water molecule. Of course the H atoms of this cannot be located but we can again see a collection of CH⋅⋅⋅O interactions with the cage interior surface with H⋅⋅⋅O contacts in the 2.6‐3 Å range; there are also some CH⋅⋅⋅O contacts between the water molecule and the 2‐adamantanone guest. As the guests show twofold positional disorder of the 2‐adamantanone and water guests (either guest could occupy either site), in accordance with the twofold symmetry of the host, it is not appropriate to over‐analyse these interactions. However it is clear that—in common with several previous examples—in complex **H⋅1** there is one guest and one water molecule in the cavity, with the guest positioned such that its carbonyl group is docked into one of the H‐bond donor pockets, lying 5.59 Å from the nearby Co^II^ centre at the back of the pocket ‐comparable to what we have seen in previous examples. This Co(cage)⋅⋅⋅O(guest) separation provides a convenient measure of the proximity of the H‐bond acceptor atom on the guest to the docking site on the host.

**Figure 3:**
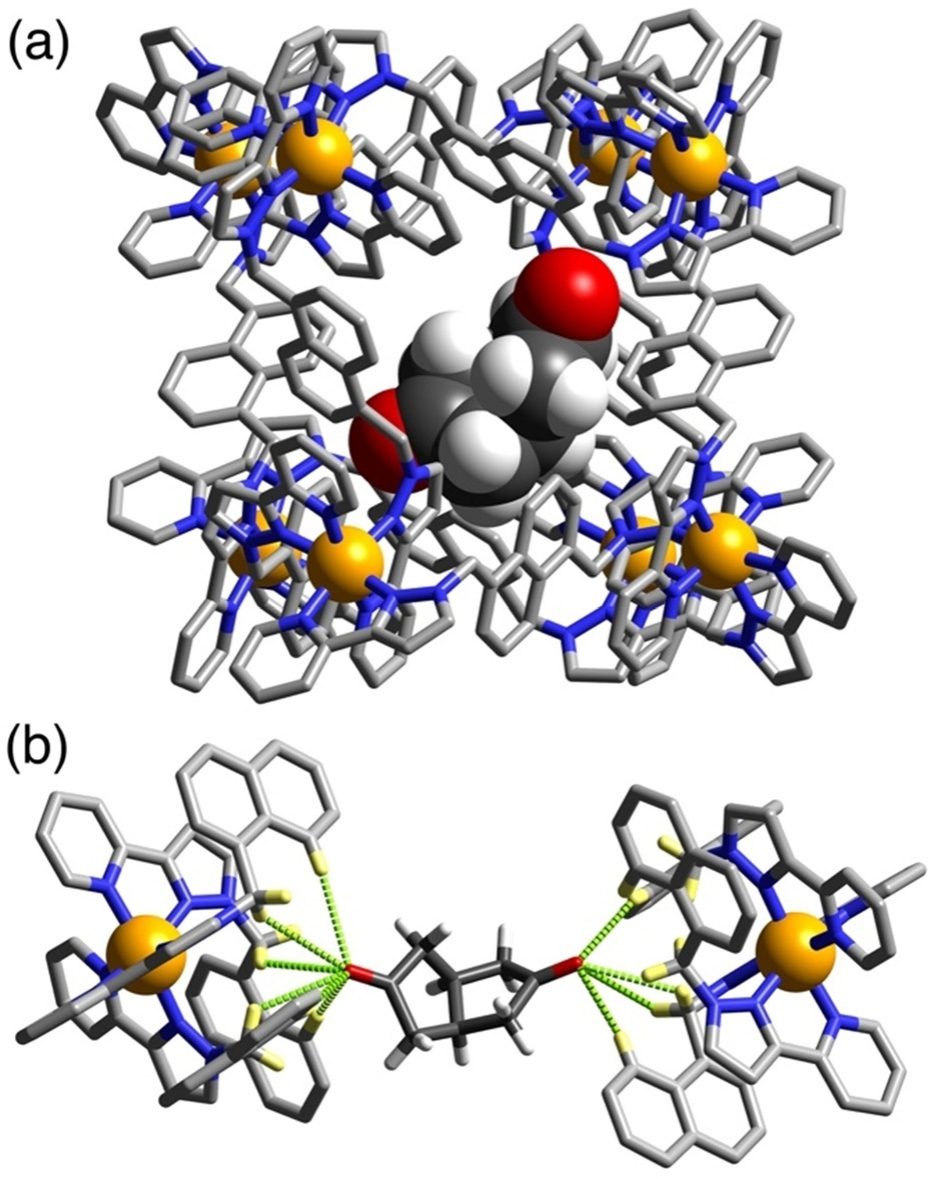
(a) Structure of the complex **H⋅2** with the host cage **H** in wireframe but the guest **2** shown space‐filling. (b) H‐bonding interactions in the structure of the **H⋅2** complex between the carbonyl unit of the guest and the two H‐bond donor pockets on the host. CH⋅⋅⋅O distances, 2.53–2.96 Å).

The complex containing the diketone guest *cis*‐bicyclo[3.3.0]octane‐3,7‐dione (**2**) is shown in Figure 3. This guest is based on a core of two fused cyclopentyl rings with a carbonyl group at each terminus; given its size it offers the possibility to lie along the cage cavity diagonal and span the gap between the two H‐bond donor pockets, with a carbonyl group in each pocket, and this is what we observed. Each terminus of the guest shows a similar collection of CH⋅⋅⋅O interactions with its binding pocket as we saw in the previous cage, but with Co⋅⋅⋅O separations of 5.59 and 6.10 Å indicating the non‐symmetric disposition of the guest in the cavity. The curvature of the guest means that it is not symmetrically disposed through the centre of the cage cavity but lies to one side, and is thus disordered over two symmetry‐equivalent orientations—across the inversion centre—of which one is shown in Figure 3. In addition, three molecules of **2** are located outside the cavity in the spaces between host cages, with these fractional site occupancies amounting to a total of 2.25 additional molecules per cage. Along with the guest 1,2,4,5‐tetracyanobenzene,8e this is only the second example of a bifunctional guest which spans the cavity and is anchored to the cage interior surface using both H‐bonding pockets and illustrates how the arrangement of two H‐bond donor pockets can organise a bifunctional guest of appropriate dimensions inside the cavity.

### Cage/guest complexes: 1:2 binding with planar aromatic guests

In this section is described a set of related structures in which, unexpectedly, we observe a stacked pair of two planar bicyclic guests bound in the cage cavity. This came as a surprise as we had had no previous suggestions from solution binding studies that two guests could bind, with spectroscopic titrations in solution using guests of this type affording binding curves which could be fitted to 1:1 binding models from which the relevant association constants were derived. Systematic structural variations amongst the members of the guest series used have allowed us to determine any trends resulting in the structures observed. Initially we studied a series of coumarins of increasing molecular volume to examine any effects on complex structures in the solid state associated with guest size in the way that we have observed in solution. This set of guests consisted of coumarin (**3**), 4‐methyl‐coumarin (**4**), 4‐methoxycoumarin (**5**) and 7‐amino‐4‐methyl‐coumarin (**6**).

**Figure 4:**
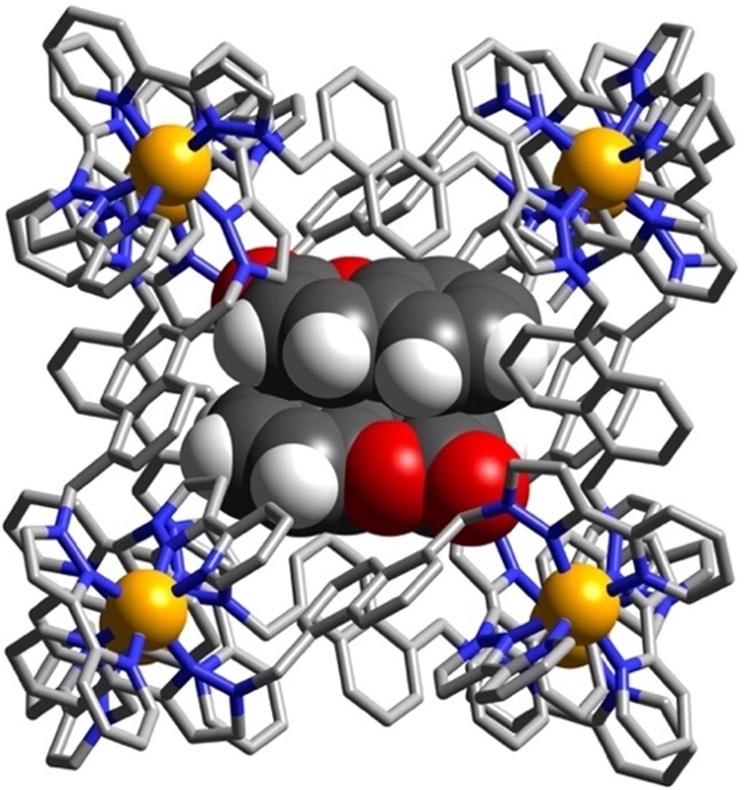
Structure of the complex **H⋅**(**3**)_2_ with the host cage **H** in wireframe but the stacked pair of guests **3** shown space‐filling.

**Figure 5:**
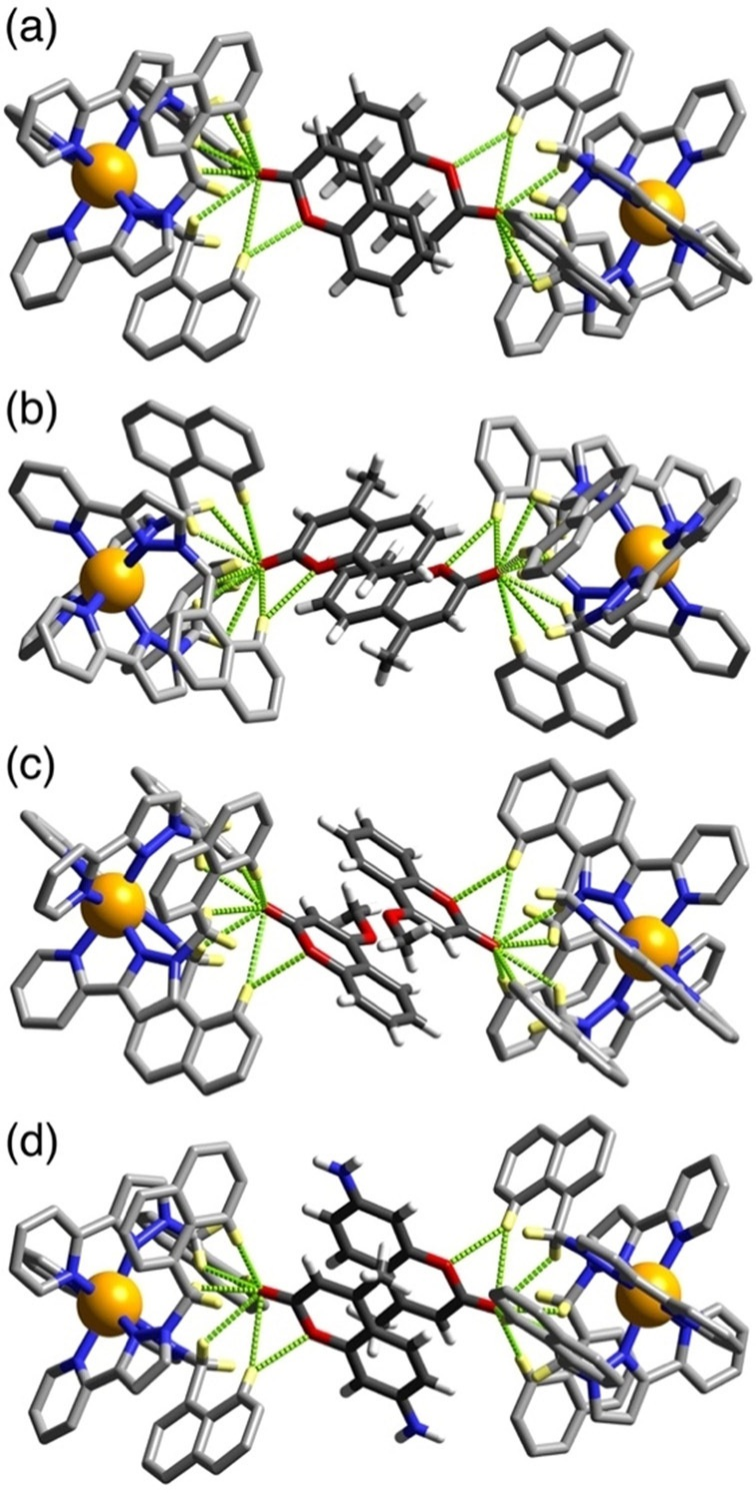
Views of parts of the structures of the **H⋅G_2_** complexes of **H** with guests (a) **3**, (b) **4**, (c) **5** and (d) **6**, showing in each case the H‐bonding interactions between the carbonyl unit of each guest and the H‐bond donor pockets on the host. CH⋅⋅⋅O distances in the H‐bonding pockets span the range 2.46–2.92 Å (for **3**, major disorder component); 2.42–2.98 Å (for **4**, major disorder component); 2.50–2.80 (for **5**, major disorder component); 2.48–42,83 Å (for **6**). In every case the two guests lie astride an inversion centre.

The structure of the complex **H⋅**(**3**)_2_ is shown in Figure 4 and 5a, and is representative of the set. A stacked pair of crystallographically equivalent guests lies either side of the inversion centre separated by a typical π‐stacking distance (separations of atoms in one molecule to the mean plane of the other is 3.3–3.4 Å). This guest pair lies in two different orientations with only the major component shown in Figure 4. In both orientations of the guest pair the exocyclic carbonyl O atom of the guest is directed into the H‐bonding pocket with the usual collection of CH⋅⋅⋅O interactions, of which the shortest are <2.5 Å; the other O atom in the coumarin ring also forms a CH⋅⋅⋅O interaction with a naphthyl CH proton that is part of the binding pocket (Figure 5 a). The Co⋅⋅⋅O separations associated with the two different orientations of the coumarin pair are 5.42 and 5.18 Å. In addition, another coumarin molecule was observed in the lattice outside the cages, in the space between cage complex units (site occupancy 0.75).

The molecular volume of coumarin is 149 Å^3^, which is 36 % of the cage cavity volume. Thus, the volume of a pair of such guests exceeds the limit suggested by Rebek of 55±9 % for the guest volume which would give optimal binding.^16^ This assumes the existence of solution equilibrium conditions. A crystalline sponge experiment such as this, in contrast, is performed under non‐equilibrium conditions with crystalline cage, containing only solvent molecules, treated with a large excess of guest: effectively, highly forcing conditions. We note also that cavity occupancy values of much higher than 55 % (occasionally >80 %) are possible if there are favourable interactions between guests, or between guest and host, which diminish the volume that they take up:^17^ and in this case we have not only obvious π‐stacking between the two guests, but each guest also forms several hydrogen bonds to the host interior surface (Figure 5 a). More generally, the difference between the pictures of guest binding shown by solution titrations and by X‐ray crystallography studies is the key point of this paper which we will return to later.

Similar behaviour is shown with the related guests 4‐methyl‐coumarin (**4**) and 4‐methoxy‐coumarin (**5**) (Figure 5). In both cases there is a parallel pair of guests stacked either side of an inversion centre with a graphitic stacking distance of 3.3–3.4 Å between the two guests; and in both cases the pair is disordered over two orientations in the cavity with the exocyclic O atoms involved in the H‐bonding “docking“ having relatively invariant positions between the two different orientations. The molecular volumes of **4** and **5** are 167 Å^3^ and 176 Å^3^ respectively, resulting in packing coefficients of 82 and 86 %, respectively, of the host cavity volume—exceptionally high values that are close to the limit of what is known.^17^

4‐Methyl‐7‐aminocoumarin (**6**) was used in solution experiments on guest binding in this family of hosts as a fluorescent reporter which was quenched on being taken up into the Co_8_ cage; displacement by competing guests restored its fluorescence, to an extent depending on the binding constant of the competing guests, and we have exploited this as the basis of a fluorescence displacement assay.8a The quenching of **6** on being taken up into the cage in water followed a 1:1 binding model and yet we see in the solid‐state, again, a stacked pair of guests in the **H⋅(6)_2_** structure (Figure 5 d). In this case the guest pair (combined volume 87 % of cavity volume) exhibits no positional disorder in the cage cavity but is confined to a single orientation, possibly because the more elongated shape of the molecule arising from the terminal amino group limits its freedom to move in the cavity. The exocyclic carbonyl O atom again shows the usual collection of weak CH⋅⋅⋅O interactions around the binding pocket, with the non‐bonded Co⋅⋅⋅O separation at the shorter end of the range that we observe (5.09 Å). This is because the CH⋅⋅⋅O separations are slightly shorter, on average, than we observed with the other coumarin‐based guests such that the carbonyl group penetrates more deeply into the binding pocket: this can be reasonably ascribed to (i) the more electron‐rich nature of this guest compared to the others due to the presence of the amine substituent; and (ii) the more elongated shape of this guest than the others. Significantly it is the carbonyl group of this guest that docks into the H‐bond donor pocket and not the amino group, indicating that the carbonyl terminus is the region of greatest electron density. However the amine N atom of guest **6** is involved in a CH⋅⋅⋅N contact (N⋅⋅⋅H distance 2.77 Å) with a CH proton of a pyrazole ring that is directly coordinated to a Co^II^ ion, indicative of the N atom acting as a weak H‐bond acceptor. Hydrogen‐bonding interactions between **6** and the host surface at both termini of the guest will contribute to the high cavity occupancy observed of 87 %, which is the largest value for any **H⋅G_2_** structure in this paper.

### Cage/guest complexes: Effects of increasing saturation

**Figure 6:**
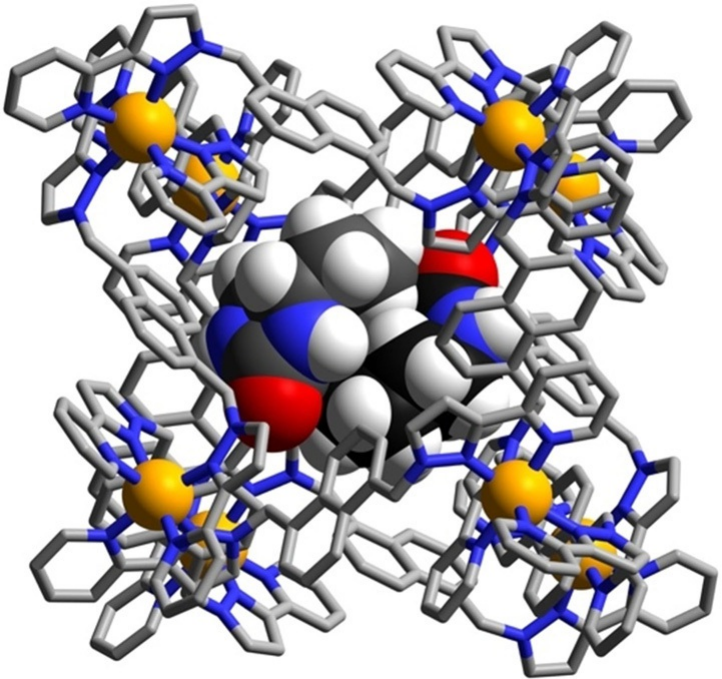
Structure of the complex **H⋅**(**9**)_2_ with the host cage **H** in wireframe but the pair of guests **9** shown space‐filling.

**Figure 7:**
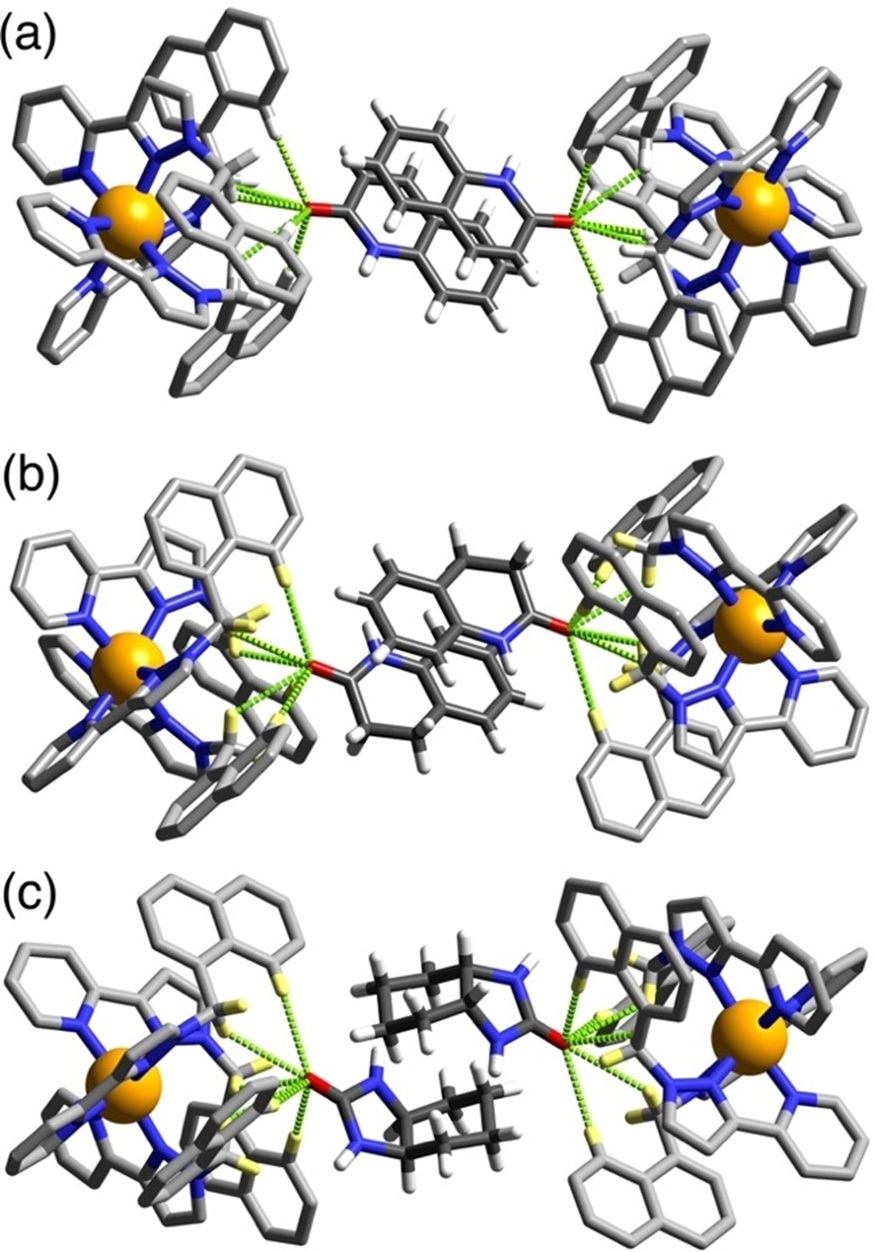
Views of parts of the structures of the **H⋅G_2_** complexes of **H** with guests (a) **7**, (b) **8**, and (c) **9**, showing in each case the H‐bonding interactions between the carbonyl unit of each guest and the H‐bond donor pockets on the host. In every case the two guests lie astride an inversion centre. CH⋅⋅⋅O distances in the H‐bonding pockets span the range 2.49–2.76 Å (for **7**); 2.48–2.79 Å (for **8**); and 2.60–2.85 Å (for **9**).

In the above series the high packing coefficients associated with having two guests in the cage cavity can be attributed in part to the forcing, non‐equilibrium conditions used for the crystalline sponge experiments. An additional factor may be the attractive aromatic stacking interaction between the pairs of guests in all members of this series, which makes the guest array more compact than would be expected for two non‐interacting molecules in solution. To see if this π‐stacking between planar aromatic guests is a prerequisite for the incorporation of two guests in the solid state we examined the structures of some complexes with bicyclic guests **7**–**9** having increasing amounts of saturation in the skeleton (Figure 6, Figure 7).

Starting with 2‐quinolinone (**7**) we have a guest that is essentially isostructural with coumarin, apart from the cyclic O being replaced by NH, and unsurprisingly this complex shows the same features as the others which contain two planar guests and an exocyclic H‐bond‐acceptor atom lying in the H‐bond donor pocket at one corner of the cage. Saturation of the double bond of the guest to generate dihydroquinolinone (**8**) has little structural effect. The arrangement of the two guests across the inversion centre is such that the aromatic ring of one guest lies parallel to and stacked with the non‐aromatic (and now non‐planar) ring of the other guest which tends to suggest that aromatic stacking is not a particularly significant issue compared to steric factors in formation of these **H⋅G_2_** complexes. This suggestion is further reinforced by the structure of the complex containing the wholly saturated guest *cis*‐octahydro‐benzimidazole‐2‐one (**9**). In this case the curvature of the guest arising from the *syn* arrangement of the two rings with respect to one another means that aromatic stacking interactions cannot be relevant in this case, and yet we still see a pair of guests occupying the cavity (packing coefficient 71 %) with each one docked into a different H‐bond donor pocket of the host, in the same way as the structures observed with the aromatic guest pairs.

The curvature of the guests **9** results in the two of them interlocking to give an approximately pseudo‐spherical assembly (Figure 6). Comparison of this with guest **2**, which is similarly curved and has approximately the same molecular volume, is interesting: only one equivalent of guest **2** binds (Figure 3) because the bifunctional nature of **2** means that it occupies both H‐bond donor pockets of the host cage simultaneously. In contrast guest **9** only interacts with one H‐bond donor pocket, meaning that a second guest can be accommodated.

### Cage/guest complexes: Other bicyclic guests

**Figure 8:**
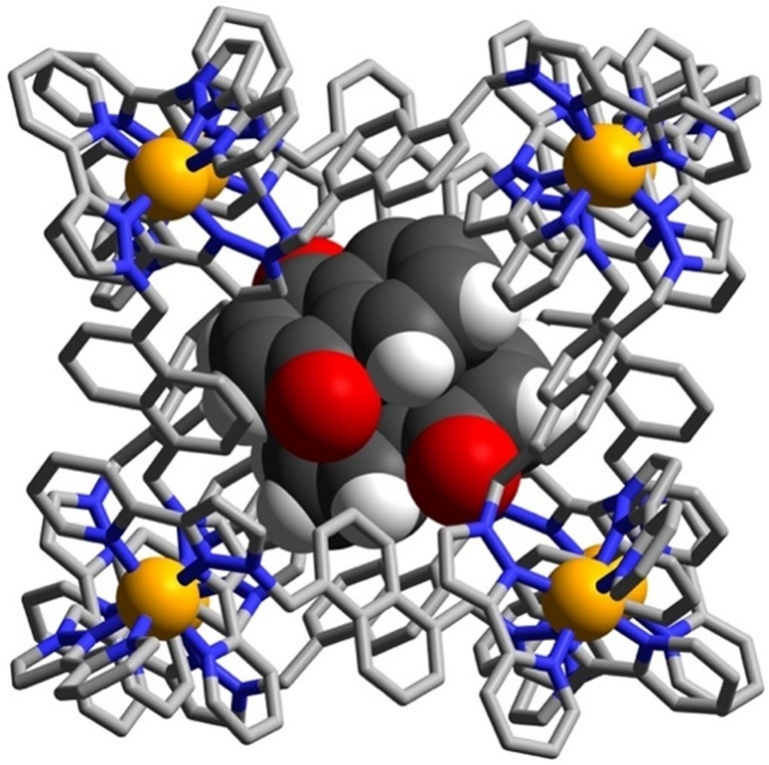
Structure of the complex **H⋅**(**12**)_2_ with the host cage **H** in wireframe but the stacked pair of guests **12** shown space‐filling.

**Figure 9:**
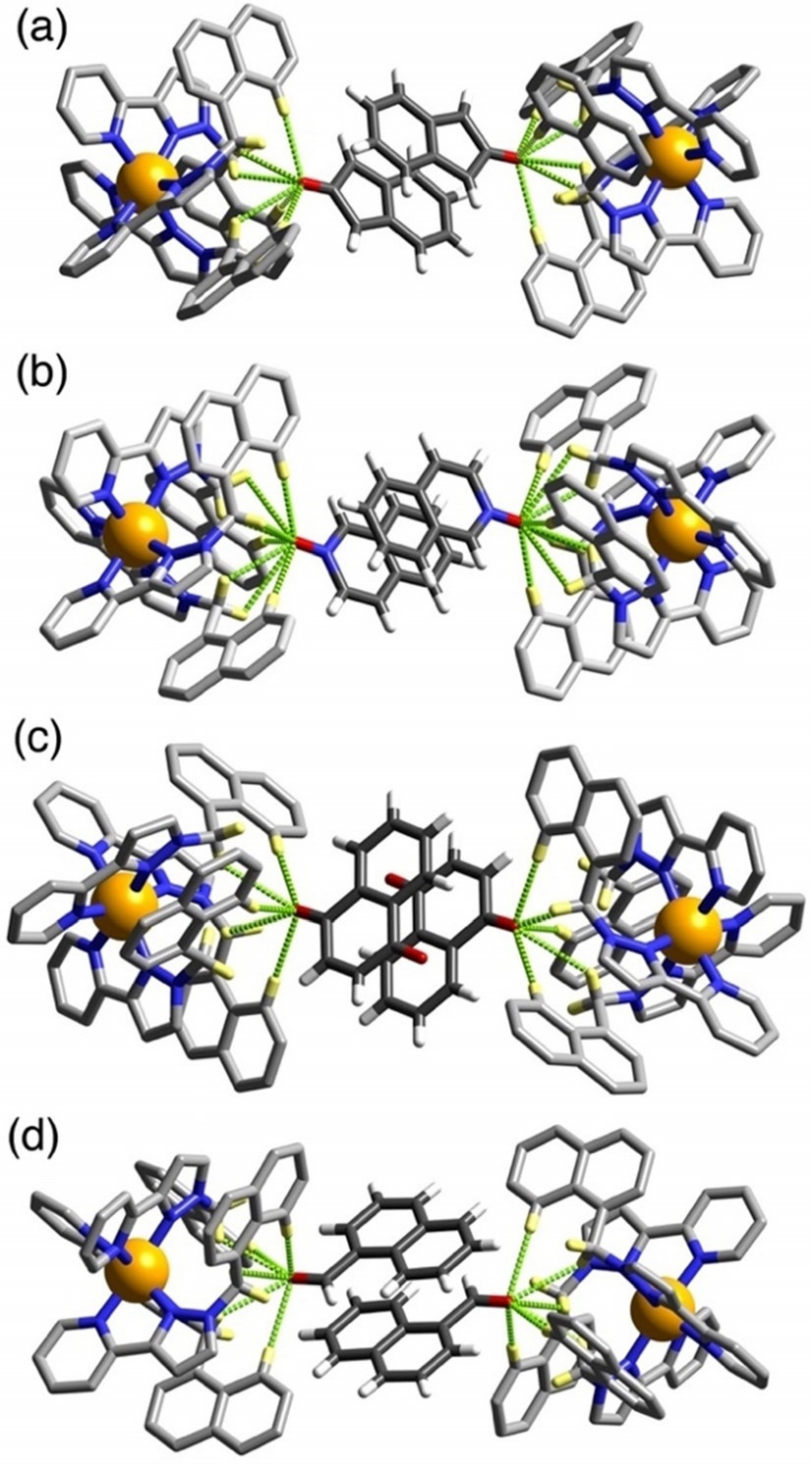
Views of parts of the structures of the **H⋅G_2_** complexes of **H** with guests (a) **10**, (b) **11**, (c) **12** and (d) **13**, showing in each case the H‐bonding interactions between the carbonyl unit of each guest and the H‐bond donor pockets on the host. In every case the two guests lie astride an inversion centre. CH⋅⋅⋅O distances in the H‐bonding pockets span the range 2.42–2.77 Å (for **10**); 2.50–2.77 Å (for **11**); 2.51–2.98 Å (for **12**); 2.56–2.84 Å (for **13**).

The similarity of all of the **H⋅G_2_** structures in this series—over a range of guest sizes and degrees of saturation—suggests that this structural behaviour is general such that other bicyclic guests of this general size and shape should give the same type of structure. This proved to be the case using the guests indan‐2‐one (**10**), isoquinoline‐*N*‐oxide (**11**; a particularly good H‐bond acceptor due to the high partial negative charge on the exocyclic oxygen atom),^7^ naphthoquinone (**12**; a quencher of the excited state in two isostructural cages which incorporate either metal‐based8e or ligand‐based8f luminophores in the superstructure), and 1‐naphthaldehyde (**13**). The disposition of the pair of guests in the cage cavity, and the main H‐bonding interactions with the H‐bond donor pockets on the cage interior surface, are summarised in Figure 8 and Figure 9.

Of these structures the one with **12** as guest (Figure 8) is noteworthy as it is bifunctional (like **2** reported above), although in this case the distance between two carbonyl groups is too short to allow the quinone unit to span the cavity and interact with both H‐bond donor pockets. However we note that one of the quinone O atoms of **12** docks with the H‐bond donor site at one of the *fac* tris‐chelate positions in the usual way, forming CH⋅⋅⋅O contacts as short as 2.51 Å with the convergent set of CH protons at that site (Figure 9 c); and the second O atom forms a particularly short CH⋅⋅⋅O interaction (O⋅⋅⋅H separation 2.36 Å) with a naphthyl CH proton associated with a different part of the cage surface. Thus, both O atoms of the guest participate in H‐bonding interactions with the host surface (*cf*. the behaviour of guest **6**), and again we see that pi‐stacked pair of guests astride an inversion centre such that the electron deficient part of one guest is parallel to and stacked with the electron rich part of the other. Packing coefficients for the cavity containing a pair of guests increase across this series from 71 % (guest **10**) to 84 % (guest **13**).

### Solution studies on guest binding

Having seen several unexpected examples of 1:2 host–guest structures in the solid state, as reported above, we were interested to revisit some solution‐based measurements of binding constants to see if we could find evidence for formation of 1:2 complexes in solution, and if so under what conditions.

In some of our earlier studies of guest binding, NMR titrations of the host cage with guests in solution sometimes showed that free and bound guest are in fast exchange on the NMR timescale: this results in a steady shift for the signal being observed as the titration proceeds, and the resulting binding curves could normally be satisfactorily fit to a 1:1 model.^6^, 8e The sole exception to this was with the small guest DMMP (mentioned earlier),^10^ for which clear evidence of a **H⋅G_2_** complex was obtained from the NMR titration. In many other cases luminescence titrations have been used, and again—with a range of guests—binding curves that fit to a 1:1 binding model were obtained.8a, 8e, 10 Thus, neither NMR nor luminescence titrations have provided—in any of our extensive previous work—any indication that guests such as those reported in this paper could form 1:2 **H⋅G_2_** complexes in solution. We note also that in some cases, titrations of the host cage with guests in solution showed by NMR spectroscopy that the guest binds in slow exchange, with separate signals observable for free and occupied cage.^6^, ^7^ In these cases binding constants are determined by integration of these separate signals, plus the knowledge of the overall concentrations of host and guest in the sample. This of course does not prove 1:1 **H⋅G** binding: in these cases, a 1:1 model was assumed and *K* values were determined on that basis.^6^ We emphasise that until the work in this paper all crystal structures of cage/guest complexes we obtained with this host (with the exception of DMMP mentioned earlier)^10^ showed incorporation of one guest.

The two guests whose binding in the cages in solution we have re‐examined are 4‐methyl‐coumarin (**4**) and 7‐amino‐4‐methyl‐coumarin (**6**). We reported the binding constant for **6** in the cavity of **H^w^** in aqueous solution a few years ago as 2.0(±0.2)×10^4^ m ^−1^, on the basis of a fluorescence titration in which the fluorescence of a fixed concentration of **6** was measured as **H^w^** was titrated in. In this experiment the normal roles of “host“ and “guest” in this titration are reversed for the spectroscopic convenience of being able to monitor quenching of a fixed amount of the guest; the fluorescence is steadily quenched by proximity to Co^II^ ions as guest **6** is taken up into the cage cavity.8a

**Figure 10:**
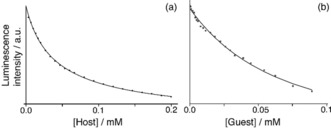
Luminescence titrations used to determine cage/guest binding constants in aqueous solution. (a) A solution of 10 μm of fluorescent guest **6** quenched by addition of increasing portions of the Co_8_ cage **H^w^** (in this titration the normal roles of ‘host“ and ‘guest” have been reversed for spectroscopic convenience). (b) A solution of 5 μm of fluorescent Cd_8_ cage **H^w^⋅Cd**, quenched on uptake of added guest **4**. In both cases good fits to a 1:1 binding model were obtained; see main text.

A repeat measurement (this work, Figure 10 a) gave a similar result with the binding constant measured this time as 3.3(±0.2)×10^4^ m ^−1^ with a good fit to a 1:1 binding curve. If we fit the data to a 1:2 binding isotherm we can obtain values of *K*_1_=6.0(±0.5)×10^4^ m ^−1^ and *K*_2_=3.6(±0.4)*×*10^3^ m ^−1^. The sum of residuals from the fit—a key element in a critical analysis of binding constants^12^—is slightly improved, as would always be expected when additional parameters allowed in the curve fitting, but there is no compelling reason to assume a speciation behaviour more complex than 1:1 in solution on the basis of this titration without additional information to support a more elaborate model.

**Figure 11:**
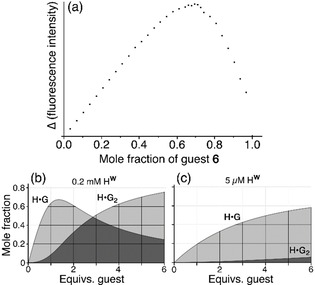
(a) Job plot obtained by combining host **H^w^** with fluorescent guest **6** in mole ratios from 1:0 to 0:1 (total concentration 0.2 mm). The *y*‐axis is the fractional decrease in luminescence at each composition compared to what would occur if all **6** were unbound, that is, it takes account of the varying amount of **6**. The maximum close to 0.7 is a clear indication of formation of the complex **H^w^⋅6_2_**, in contrast to the titration in Figure 9 a using the same components, which was consistent with 1:1 complex formation (see main text). Parts (b) and (c) show the speciation of the **H^w^⋅6_2_** system at different host concentrations, based on association constant values of *K*_1_=6×10^4^ m ^−1^ and *K*_2_=3.6×10^3^ m ^−1^ that were obtained by fitting the titration data from Figure 10 a to a 1:2 isotherm, see main text. The dominance of **H^w^⋅6_2_** at high host concentrations (Job plot), and its virtual absence at low guest concentrations (fluorescence titration), are clear.

An alternative way of evaluating complex stoichiometry is the Job plot. Concerns about the validity of these have been expressed recently as it is easy to use them inappropriately and get misleading results.^12^ Whilst use of a Job plot to confirm 1:1 complex formation is straightforward, evaluation of 1:2 **H⋅G_2_** complex formation—with the maximum in the plot at a host mole fraction of 0.33 and a guest mole fraction of 0.67—requires that binding is strong at the concentration conditions used such that the **H⋅G_2_** complex dominates solution speciation. Further complications occur if the spectroscopic changes Δ_1_ and Δ_2_ (changes in measured quantity arising from binding the first guest and then again from binding the second guest) are different.^12^ For binding of **6** inside the cavity of **H^w^** this latter issue is avoided if we assume that all bound molecules of **6** are quenched by their proximity to Co^II^ ions, such that binding the first and then the second guest result in an equal loss of luminescence intensity (Δ_1_=Δ_2_). The result of a Job plot experiment using different mole fractions of **H^w^** and **6** in water with a combined concentration of 0.2 mm is shown in Figure 11 a. The *y*‐axis shows the loss in luminescence intensity associated with guest binding. The result clearly shows a maximum at a guest mole fraction of two thirds, indicating that **H^w^⋅6_2_** complex formation dominates in solution under these conditions. This is the first clear indication we have had that any guest of this size can form an **H⋅G_2_** complex in solution with this cage.

Thus we have a situation in which the fluorescence titration curve [at 10 μm guest, Figure 10 a] fits to a 1:1 binding model, but the Job plot (at combined concentration of components of 200 μm, Figure 11 a) indicates that 1:2 **H⋅G_2_** complex formation can occur in solution—and the crystal structure confirms 1:2 **H⋅G_2_** complex formation in the solid state. The contradiction arises from the different conditions used for each experiment. For the fluorescence titration, addition of increasing amounts of cage **H^w^** to a fixed amount of fluorescent guest **6** means that the cage is in increasingly larger excess as the titration proceeds. This, plus the low concentration, results in conditions which will favour 1:1 complex formation. Given that binding of the second guest would result in >80 % cavity occupancy, it is reasonable to expect that *K*_2_≪*K*_1_. Moreover at low concentrations, simple simulations have shown that *K*_2_ can be appreciable compared to *K*_1_ and yet the binding curve, which is dominated by the first binding event, still apparently fits to a 1:1 model.^12^ The maximum in the Job plot, in contrast, was obtained at a much higher concentration (combined concentration, 0.2 mm) with proportions of components optimised for 1:2 **H⋅G_2_** complex formation. Under these conditions it is clear that *K*_2_ is large enough to allow the **H⋅G_2_** complex to dominate in the solution speciation, that is, *K*_2_>(1/concentration) which is 5000 m ^−1^. The effects of different concentrations of the titration experiment versus Job plot experiment on the solution speciation are illustrated in Figures 11 b and 11 c.

To examine binding of **4** we again started with a fluorescence titration. This time we used a fluorescent host: 5 μm of the Cd_8_ analogue of **H^w^** (denoted **H^w^⋅Cd**) which is isostructural with the Co_8_ cage **H^w^** and retains the fluorescence arising from the array of 12 naphthyl groups in the ligand set as the d^10^ Cd^II^ ion is non‐quenching.8f Addition of increasing amounts of **4** during the titration progressively quenches the fluorescence of **H^w^⋅Cd** as the guest binds (Figure 10 b). By the end of the experiment there is a 20‐fold excess of guest added (instead of the cage being in excess, as in the **H^w^**/**6** titration) which will maximise the likelihood of observing any **H⋅G_2_** complex in the later stages of the titration. However, analysis of the binding curve again revealed—after multiple repeat experiments—that it fitted well to a 1:1 binding model with *K*_1_=2.1(±0.2)×10^4^ m ^−1^. Allowing a second binding constant in the fitting afforded *K*_1_=1.7(±0.2)×10^4^ m ^−1^ and *K*_2_=4.5(3.1)×10^3^ m ^−1^ with a very large error on *K*_2_ and again not an obvious improvement in residuals. From a conventional titration there is, again, no reason to assume anything other than 1:1 **H⋅G** binding under these conditions at which, if *K*_2_≪*K*_1_, the curve shape is dominated by the first binding event.

There is an additional experimental limitation in this titration experiment that was not present with the previous example (**H^w^**/**6**). If the fluorescence of **H^w^⋅Cd** is substantially (or fully) quenched by binding of the first guest molecule **4**, then binding of a second equivalent of **4** will cause little (or no) further change in the quantity being measured; that is, Δ_1_≫Δ_2_. This would make the shape of the binding curve insensitive to *K*_2_ and result in the data being consistent with 1:1 binding, as observed. Another consequence of this is that the limited conditions that make a Job plot a legitimate way of analysing the stoichiometry of the system are not met here: if Δ_1_≫Δ_2_ then a reliable result from a Job plot analysis is not to be expected.^12^ We arrive at the conclusion that the fluorescence titration gives an incomplete picture, with the curve being dominated by the first binding event not just because of the low concentration but also because Δ_2_≪Δ_1_: and a Job plot will also not be a reliable indicator of stoichiometry.

**Figure 12:**
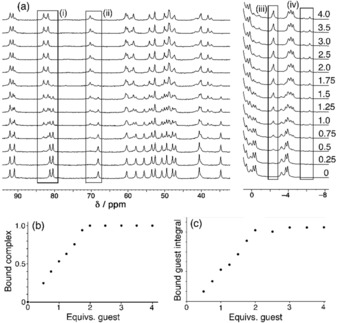
An NMR titration performed by addition of up to four equivalents of guest **4** to the Co_8_ cage **H^w^** (0.15 mm, D_2_O, 298 K). (a) Stacked plot showing evolution of the spectra as guest is added (number of equivalents of added guest is indicated). Boxes (i) and (ii) illustrate how the signals for empty cage (bottom spectrum) are replaced by new signals for bound cage (top spectrum): integration of these allows the fraction of cage occupied by guest to be determined in each spectrum. Boxes (iii) and (iv) illustrate the grow‐in of signals for the bound guest in the paramagnetic cavity: integration of these allows a separate measurement of the fraction of cage occupied by guest during the titration. (b) Graph of fraction of complex containing guest (from 0 to 1) based on integration of free/ bound cage signals at 68–70 ppm [box (ii) in part (a)]. (c) Graph of magnitude of integral for bound guest signal at −2.3 ppm [box (iii) in part (a)]. Both graphs (b) and (c) confirm strong binding of two guests under these conditions, see main text.

We therefore performed an NMR titration at higher concentrations. The low solubility of the cage **H^w^⋅Cd** compared to its Co_8_ analogue **H^w^** (arising largely from the difference in counter‐ions) precludes this, so we changed the cage to the more soluble but isostructural **H^w^** at 0.15 mm and titrated in guest **4** up to a total of four equivalents. Figure 12 a shows the evolution of ^1^H NMR spectra during the titration. We can see how paramagnetically shifted signals for free cage **H^w^** diminish during the titration and are replaced by new signals associated with complex formation, with free and bound guests being in slow exchange on the NMR timescale. We can also see, at negative chemical shift values, new signals growing in associated with guest molecules in the cavity surrounded by eight paramagnetic high‐spin Co^II^ ions. Integration of these allows plots of proportion of bound cage vs. amount of added guest to be produced [Figure 12, parts (b) and (c)] which show a linear increase in complex formation with added guest until 2 equivalents of **4** are added, after which there is no further change and any further added guest appears in the normal aromatic region of the NMR spectrum. This is clearly consistent with two guests binding in the host cavity, with *K*_1_ and *K*_2_ being at the strong limit [≫ (1/concentration)] under these conditions.

Again, therefore—as with guest **6**—we have two experiments performed under different conditions giving different results, with the fluorescence titrations indicating 1:1 binding but the Job plot (for **6**) and the NMR experiment (for **4**) indicating that 1:2 complex formation can also occur, in agreement with the crystallographic results. Together, these observations highlight the recently‐made points about the difficulties associated with determining stoichiometries of supramolecular complexes in solution and the benefits of using different techniques to probe this under different conditions—as well as incorporating in the analysis chemical information obtained about possible host–guest ratios from other sources (such as X‐ray crystallography).^11^, ^12^

## Conclusions

A crystallographic investigation of an extensive series of host–guest complexes based on the octanuclear cubic coordination cage host **H** revealed a range of different guest binding motifs. 1:1 **H⋅G** complexes were formed in which a mono‐ketone (2‐adamantanone, **1**) and a di‐ketone (*cis*‐bicyclo[3.3.0]octane‐3,7‐dione, **2**) were bound in the cage cavity, anchored by H‐bonding interactions between the ketone group on the guest and one or two hydrogen‐bond‐donor sites on the cage interior surface. In the latter case the distance between the two ketone groups in the guest is fortuitously appropriate to allow this guest to span the long diagonal of the cube host and interact with both H‐bond donor sites simultaneously.

With many other bicyclic guests however (**3**–**13**), including a family of substituted coumarins as well as some saturated bicyclic analogues, we unexpectedly observed in the crystal structures the inclusion of two guests in the cage cavity lying astride an inversion centre. In every case each guest showed the same type of hydrogen‐bonding interaction with the pockets on the cage interior surface that we saw in the 1:1 complexes, with each guest interacting with one of the two binding pockets. For the aromatic members of this series the two guests are separated by a graphitic π‐stacking distance; however, with saturated analogues the cavity still accommodates two guests, indicating that π‐stacking in the pair is not a prerequisite for both guests to fit in the cavity. The sum of the molecular volumes of the two guests—up to 87 % of the cavity volume, which are some of the highest packing coefficients known^17^—substantially exceeds the Rebek 55±9 % limit for optimal guest binding in a cavity in solution. We ascribe this to two factors: (i) our experiments were based on a ‘crystalline sponge“ methodology using a large excess of guest being soaked into crystals of empty host molecules under non‐equilibrium conditions; and (ii) the combination of favourable guest‐guest interactions (π‐stacking in many cases) and favourable guest–host interactions (H‐bonding between guest and the cage interior surface) combine to produce a particularly compact guest array which nicely matches the cage cavity shape.

Given the knowledge that 1:2 **H⋅G_2_** complex formation is possible in the solid state, we could find evidence for it also happening in solution with experiments designed to optimise this behaviour. Whilst conventional fluorescence titrations with guests **4** and **6** both gave binding curves that could be fitted to 1:1 binding, experiments at higher concentration—a Job plot with guest **6** (under conditions where this is legitimate^12^), and an NMR titration using guest **4**, both confirmed that **H⋅G_2_** complex formation can also occur in solution. Importantly however these experiments were done based on the separate knowledge from crystallography that **H⋅G_2_** complex formation was feasible at all, which was not expected on the basis of guest sizes, and not apparent from conventional titration data. We note that Heitz and co‐workers have likewise recently reported a porphyrin‐based cage structure which accommodates a stacked pair of aromatic guests in the solid state (giving a high packing coefficient of 84 %), despite solution measurements indicating 1:1 host–guest binding.17c

This is potentially interesting as encapsulation of two guests in the confined space of a cage cavity, even if this constitutes only a small proportion of the equilibrium speciation in solution, could potentially provide a pathway to new forms of catalytic behaviour or altered reactivity associated with a pair of molecules held in close proximity. There are many examples of light‐triggered reactions between two species held in close proximity in supramolecular assemblies—either cage‐type species,^18^ or coordination networks^19^—as well as examples of reactions between two co‐encapsulated guests which are catalysed, or occur with altered regioselectivity, because of the steric or electronic properties of the host.^20^ The observations reported in this paper suggest that the catalytic properties associated with the **H**/**H^w^** cage system^3^ could be extended in these directions.

Overall, these results provide substantially improved insights onto the guest binding and potential future catalysis‐based properties of our cage system **H**/**H^w^**: and also highlight some of the recently expressed difficulties in determining stoichiometries of supramolecular complexes.^12^

## Experimental Section

Batches of single crystals of **H** used for the X‐ray diffraction experiments were prepared solvothermally from a mixture of Co(BF_4_)_2_ and the ligand **L** in a 2:3 ratio in MeOH using the method previously published.^5^ Crystals were screened using an optical microscope and good‐quality ones were selected for crystalline sponge experiments, which involved immersing the crystal either in pure guest (if it is an oil), or in a concentrated MeOH solution of the guest, for 2 days. Crystals were transferred to Fomblin oil before being mounted on a MiteGen Microloop and flash frozen and stored in liquid nitrogen.

X‐ray crystallography measurements were performed in Experiment Hutch 1 of beamline I‐19 at the UK Diamond Light Source synchrotron facility,^21^ using the automatic sample‐changing robot.^22^ The data were collected at a wavelength of 0.6889 Å on a Fluid Film Devices 3‐circle fixed‐chi diffractometer using a Dectris Pilatus 2 m detector. Each crystal was mounted on a MiTeGen micromount using a perfluoropolyether oil, and cooled for data collection by a Cryostream nitrogen‐gas stream.^23^ The collected frames were integrated using DIALS software^24^ and the data were corrected for absorption effects using AIMLESS, an empirical method.^25^ The structures were solved by dual‐space methods,^26^ and refined by least‐squares refinement on all unique measured *F* ^2^ values.^26^ A summary table of crystallographic and data collection parameters and CCDC deposition numbers is provided in the Supporting Information. Of the complexes containing a pair of guests (**3**–**13**), most of the crystal structures refined successfully with each guest having 100 % site occupancy in the asymmetric unit, that is, there are two complete guests per cage. The exceptions are guest **9** which refined with a site occupancy of 0.63 per asymmetric unit (i.e. 1.26 guests per cage), and guest **12** which refined with a site occupancy of 0.86 per asymmetric unit (i.e. 1.72 guests per cage), presumably due to incomplete uptake of guest by the crystal used. However the total occupancy of >1 guest per cavity in each case confirms the possibility for two guests to be bound simultaneously.

Software used: binding constants were calculated using the Bindfit software, and the simulations in Figure 11 were determined using the Bindsim software, both from the website http://supramolecular.org.^27^ Molecular volumes of guests (Scheme 1), and the host cavity volume (Figure 1 b), were calculated using SPARTAN18.^28^

## Conflict of interest

The authors declare no conflict of interest.

## Supporting information

As a service to our authors and readers, this journal provides supporting information supplied by the authors. Such materials are peer reviewed and may be re‐organized for online delivery, but are not copy‐edited or typeset. Technical support issues arising from supporting information (other than missing files) should be addressed to the authors.

Supplementary
