# Supplementary material for: One Guest or Two? A Crystallographic and Solution Study of Guest Binding in a Cubic Coordination Cage
Source: Chemistry. 2020 Feb 6;26(14):3054–64. doi: 10.1002/chem.201905499 (PMC7079040; doi:10.1002/chem.201905499)
Supplement: Supplementary file 1 — Supplementary [file CHEM-26-3054-s001.pdf]

CHEMISTRY
A European Journal

Supporting Information

One Guest or Two? A Crystallographic and Solution Study of Guest Binding in a Cubic Coordination Cage

Christopher G. P. Taylor,[a] Stephen P. Argent,[a] Michael D. Ludden,[a] Jerico R. Piper,[a] Cristina Mozaceanu,[a] Sarah A. Barnett,[b] and Michael D. Ward*[a]

chem_201905499_sm_miscellaneous_information.pdf

Crystallographic data

A brief summary of data collection methodology at the UK Diamond Synchrotron is given in the main text. All structure determinations suffered from the usual weak scattering characteristic of crystals of this type, associated with large unit cells and disorder of solvents / anions. This necessitated extensive use of restraints during the refinements to achieve stable and chemically reasonable models. Some anions / solvents were refined with fractional occupancies over two sites when the disorder could be modelled. Large solvent-accessible voids containing diffuse electron density that could not be satisfactorily modelled were accounted for using the SQUEEZE command in PLATON. Full details of the treatments of the three structures, including software used, are given in the individual CIFs

CCDC deposition numbers:

01:	CCDC-1970074	08:	CCDC-1970197
02:	CCDC-1970073	09:	CCDC-1970198
03:	CCDC-1970072	10:	CCDC-1970199
04:	CCDC-1970075	11:	CCDC-1970200
05:	CCDC-1970071	12:	CCDC-1970196
06:	CCDC-1970195	13:	CCDC-1970201
07:	CCDC-1970076		

These data can be obtained free of charge from The Cambridge Crystallographic Data Centre via www.ccdc.cam.ac.uk/data_request/cif.

Table S1: Single Crystal X-ray studies – crystallographic and data collection details for complex H•[Guest]$_x$ for guests 01-04

Identification code	01_DMWMSv1-11_sq	02_DMWMSv6-15_sq	03_DMWMSv7-214_sq	04_DMWMSv2-64_sq
Empirical formula	$C_{421.5}H_{472.5}B_{13.5}Cl_{2.5}Co_8F_{54}N_{72}O_{21.75}$	$C_{385}H_{388.5}B_{14}Cl_2Co_8F_{56}N_{72}O_{29.5}$	$C_{395.5}H_{407.5}B_{16}Co_8F_{64}N_{75.5}O_{31.5}$	$C_{390}H_{416}B_{16}Co_8F_{64}N_{72}O_{38}$
Formula weight	8627.19	8253.83	8582.85	8580.32
Temperature/K	100(1)	100.0(10)	100(1)	293(2)
Crystal system	monoclinic	monoclinic	monoclinic	monoclinic
Space group	*C*2/c	*C*2/c	*C*2/c	*C*2/c
a/Å	33.20466(11)	33.4404(3)	32.92599(9)	32.94575(7)
b/Å	29.64323(11)	29.6306(2)	30.24214(8)	30.02245(7)
c/Å	40.54571(15)	40.5979(4)	40.42029(13)	40.78862(10)
α/°	90	90	90	90
β/°	95.3813(3)	95.4263(9)	95.6031(3)	96.7986(2)
γ/°	90	90	90	90
Volume/Å^3	39732.98(17)	40046.6(5)	40056.30(15)	40060.83(11)
Z	4	4	4	4
ρ_{calc} / g cm^{-3}	1.442	1.369	1.423	1.423
μ/mm^{-1}	0.413	0.406	0.396	0.4
F(000)	17966	17050	17732	17744
Crystal size/mm^3	0.1 × 0.1 × 0.08	0.1 × 0.09 × 0.09	0.15 × 0.15 × 0.14	0.06 × 0.05 × 0.04
Radiation / Å	Synchrotron (λ = 0.6889)	Synchrotron (λ = 0.6889)	Synchrotron (λ = 0.6889)	Synchrotron (λ = 0.6889)
2Θ /°	1.984 to 59.894	1.784 to 59.894	1.962 to 59.892	1.95 to 72.166
Index ranges	-48 ≤ h ≤ 48, -42 ≤ k ≤ 42, -58 ≤ l ≤ 58	-48 ≤ h ≤ 48, -42 ≤ k ≤ 42, -58 ≤ l ≤ 58	-47 ≤ h ≤ 47, -43 ≤ k ≤ 43, -58 ≤ l ≤ 58	-53 ≤ h ≤ 54, -48 ≤ k ≤ 50, -68 ≤ l ≤ 66
Reflections collected	341140	334868	350035	440028
Independent reflections	63127 [R_{int} = 0.0581, R_{sigma} = 0.0577]	63736 [Rint = 0.0776, Rsigma = 0.0788]	63790 [R_{int} = 0.0472, R_{sigma} = 0.0499]	97012 [R_{int} = 0.0554, R_{sigma} = 0.0673]
Data/restraints /parameters	63127/6590/2479	63736/6392/2548	63790/7160/2657	97012/6674/2535
Goodness-of-fit on F^2	0.937	1.062	1.096	0.941
Final R indexes [$I \geq 2\sigma(I)$]	R_1 = 0.0726, wR_2 = 0.2288	R_1 = 0.1050, wR_2 = 0.3165	R_1 = 0.0877, wR_2 = 0.2908	R_1 = 0.0732, wR_2 = 0.2393
Final R indexes [all data]	R_1 = 0.1182, wR_2 = 0.2535	R*1* = 0.1875, wR_2 = 0.3666	R_1 = 0.1259, wR_2 = 0.3203	R_1 = 0.1389, wR_2 = 0.2768
Largest diff. peak/hole / e Å^{-3}	1.84/-0.52	1.50/-0.70	1.97/-0.77	1.56/-0.53

Table S2: Single Crystal X-ray studies – crystallographic and data collection details for complex H•[Guest]$_x$ for guests 05-07

Identification code	05_DMWMSv2-56_sq	06_DMWMSv1-07_sq	07_DMWMSv6-16_sq
Empirical formula	$C_{389}H_{412}B_{16}Co_8F_{64}N_{72}O_{39}$	$C_{386}H_{410}B_{14.5}Cl_{1.5}Co_8F_{58}N_{74}O_{34}$	$C_{389.25}H_{419}B_{14.5}Cl_{1.5}Co_8F_{55.45}N_{74}O_2$
Formula weight	8580.28	8413.21	7900.96
Temperature/K	100.0(10)	100(1)	100(1)
Crystal system	monoclinic	monoclinic	monoclinic
Space group	*C*2/c	*C*2/c	*C*2/c
a/Å	33.0402(4)	32.8460(3)	32.92772(8)
b/Å	30.5110(5)	30.3426(3)	30.19467(7)
c/Å	40.5996(6)	40.5879(5)	39.93606(11)
α/°	90	90	90
β/°	96.1366(12)	96.3938(9)	96.0901(2)
γ/°	90	90	90
Volume/Å^3	40693.4(7)	40199.6(5)	39482.00(12)
Z	4	4	4
ρ_{calc} / g cm^{-3}	1.401	1.39	1.329
μ/mm^{-1}	0.394	0.404	0.4
F(000)	17736	17408	16406
Crystal size/mm^3	0.2 × 0.1 × 0.1	0.09 × 0.08 × 0.08	0.12 × 0.09 × 0.08
Radiation / Å	Synchrotron (λ = 0.6889)	Synchrotron (λ = 0.6889)	Synchrotron (λ = 0.6889)
2Θ range for data collection/°	1.956 to 59.894	1.776 to 59.894	1.778 to 59.894
Index ranges	-47 ≤ h ≤ 47, -44 ≤ k ≤ 44, -58 ≤ l ≤ 58	-47 ≤ h ≤ 47, -43 ≤ k ≤ 43, -58 ≤ l ≤ 58	-47 ≤ h ≤ 47, -43 ≤ k ≤ 43, -57 ≤ l ≤ 57
Reflections collected	264050	343505	343861
Independent reflections	64773 [R_{int} = 0.0636, R_{sigma} = 0.0734]	63984 [R_{int} = 0.1133, R_{sigma} = 0.0774]	62863 [R_{int} = 0.0590, R_{sigma} = 0.0575]
Data/restraints/parameters	64773/7321/2685	63984/5782/2305	62863/6031/2423
Goodness-of-fit on F^2	1.007	1.028	1.122
Final R indexes [$I \geq 2\sigma(I)$]	R_1 = 0.0817, wR_2 = 0.2640	R_1 = 0.0998, wR_2 = 0.3051	R_1 = 0.0626, wR_2 = 0.2052
Final R indexes [all data]	R_1 = 0.1314, wR_2 = 0.3037	R_1 = 0.1721, wR_2 = 0.3526	R_1 = 0.0890, wR_2 = 0.2201
Largest diff. peak/hole / e Å^{-3}	1.22/-0.75	1.73/-0.62	1.35/-0.79

Table S3: Single Crystal X-ray studies – crystallographic and data collection details for complex H•[Guest]$_x$ for guests 08-10

Identification code	08_DMWMSv1-04_sq	09_DMWMSv1-09_sq	10_DMWMSv1-03_sq
Empirical formula	$C_{390}H_{426}B_{14}Cl_2Co_8F_{56}N_{74}O_{38}$	$C_{380.25}H_{407.25}B_{14.5}Cl_{1.5}Co_8F_{58}N_{73.25}O_{34.25}$	$C_{392}H_{432}B_{13.5}Cl_{2.5}Co_8F_{54}N_{72}O_{40}$
Formula weight	8515.7	8334.88	8524.07
Temperature/K	100(1)	100(1)	100(1)
Crystal system	monoclinic	monoclinic	monoclinic
Space group	*C*2/c	*C*2/c	*C*2/c
a/Å	32.86138(16)	33.0544(3)	32.87015(19)
b/Å	29.99192(14)	29.84286(19)	29.94363(17)
c/Å	40.4007(2)	40.5425(4)	40.4002(3)
α/°	90	90	90
β/°	96.1437(5)	96.8175(7)	96.1870(5)
γ/°	90	90	90
Volume/Å^3	39589.2(3)	39709.9(4)	39532.3(3)
Z	4	4	4
ρ_{calc} / g cm^{-3}	1.429	1.394	1.432
μ/mm^{-1}	0.415	0.408	0.418
F(000)	17648	17246	17680
Crystal size/mm^3	0.1 × 0.08 × 0.06	0.15 × 0.13 × 0.08	0.09 × 0.08 × 0.07
Radiation / Å	Synchrotron (λ = 0.6889)	Synchrotron (λ = 0.6889)	Synchrotron (λ = 0.6889)
2Θ range for data collection/°	1.966 to 59.894	1.788 to 59.894	1.788 to 59.894
Index ranges	-47 ≤ h ≤ 47, -43 ≤ k ≤ 43, -58 ≤ l ≤ 58	-47 ≤ h ≤ 47, -43 ≤ k ≤ 43, -58 ≤ l ≤ 58	-47 ≤ h ≤ 47, -43 ≤ k ≤ 43, -58 ≤ l ≤ 58
Reflections collected	343759	337675	344085
Independent reflections	62994 [R_{int} = 0.0632, R_{sigma} = 0.0647]	63142 [R_{int} = 0.0604, R_{sigma} = 0.0530]	62949 [R_{int} = 0.0634, R_{sigma} = 0.0632]
Data/restraints/parameters	62994/6619/2459	63142/6379/2415	62949/6167/2374
Goodness-of-fit on F^2	0.945	1.006	0.958
Final R indexes [$I \geq 2\sigma(I)$]	R_1 = 0.0644, wR_2 = 0.1984	R_1 = 0.0897, wR_2 = 0.2844	R_1 = 0.0710, wR_2 = 0.2257
Final R indexes [all data]	R_1 = 0.1137, wR_2 = 0.2188	R_1 = 0.1466, wR_2 = 0.3241	R_1 = 0.1254, wR_2 = 0.2504
Largest diff. peak/hole / e Å^{-3}	1.54/-0.63	1.74/-0.47	1.67/-0.89

Table S4: Single Crystal X-ray studies – crystallographic and data collection details for complex H•[Guest]$_x$ for guests 11-13

Identification code	11_DMWMSv1-10_sq	12_DMWMSv3-153_sq	13_DMWMSv6-67_sq
Empirical formula	$C_{391}H_{426}B_{14.5}Cl_{1.5}Co_8F_{58}N_{74}O_{39}$	$C_{383.2}H_{360.32}B_{16}Co_8F_{64}N_{72}O_{25.44}$	$C_{379}H_{400}B_{16}Co_8F_{64}N_{72}O_{33}$
Formula weight	8569.39	8241.57	8352.09
Temperature/K	100(1)	100(1)	100(1)
Crystal system	monoclinic	monoclinic	monoclinic
Space group	*C*2/c	*C*2/c	*C*2/c
a/Å	32.93081(15)	32.73449(16)	33.01913(8)
b/Å	30.14323(16)	29.70761(14)	30.10586(7)
c/Å	40.1198(2)	40.27685(18)	40.54287(11)
α/°	90	90	90
β/°	96.1151(5)	95.9837(4)	96.1271(2)
γ/°	90	90	90
Volume/Å^3	39598.0(3)	38954.4(2)	40072.20(13)
Z	4	4	4
ρ_{calc} / g cm^{-3}	1.437	1.405	1.384
μ/mm^{-1}	0.413	0.403	0.397
F(000)	17752	16956	17256
Crystal size/mm^3	0.1 × 0.1 × 0.08	0.06 × 0.06 × 0.06	0.15 × 0.15 × 0.14
Radiation / Å	Synchrotron (λ = 0.6889)	Synchrotron (λ = 0.6889)	Synchrotron (λ = 0.6889)
2Θ range for data collection/°	1.974 to 59.894	1.97 to 59.894	1.778 to 59.894
Index ranges	-47 ≤ h ≤ 47, -43 ≤ k ≤ 43, -58 ≤ l ≤ 58	-36 ≤ h ≤ 47, -43 ≤ k ≤ 29, -58 ≤ l ≤ 58	-47 ≤ h ≤ 47, -43 ≤ k ≤ 43, -58 ≤ l ≤ 58
Reflections collected	339505	172603	351006
Independent reflections	62951 [R_{int} = 0.0419, R_{sigma} = 0.0394]	61172 [R_{int} = 0.0650, R_{sigma} = 0.0968]	63721 [R_{int} = 0.0584, R_{sigma} = 0.0549]
Data/restraints/parameters	62951/6175/2419	61172/6374/2531	63721/6193/2437
Goodness-of-fit on F^2	1.089	1.013	1.073
Final R indexes [$I \geq 2\sigma(I)$]	R_1 = 0.0593, wR_2 = 0.1949	R_1 = 0.0737, wR_2 = 0.2158	R_1 = 0.0785, wR_2 = 0.2641
Final R indexes [all data]	R_1 = 0.0823, wR_2 = 0.2064	R_1 = 0.1158, wR_2 = 0.2440	R_1 = 0.1091, wR_2 = 0.2901
Largest diff. peak/hole / e Å^{-3}	1.41/-0.80	1.41/-0.76	1.19/-1.03
